# Microtubules Disruption Alters the Cellular Structures and Mechanics Depending on Underlying Chemical Cues

**DOI:** 10.1002/smll.202312282

**Published:** 2024-09-29

**Authors:** Shimaa A. Abdellatef, Hongxin Wang, Jun Nakanishi

**Affiliations:** ^1^ Research Center for Macromolecules and Biomaterials National Institute for Materials Science (NIMS) 1‐1 Namiki Tsukuba 305‐0044 Japan; ^2^ Graduate School of Advanced Engineering Tokyo University of Science 6‐3‐1, Niijuku Katsushika‐ku Tokyo 125‐8585 Japan; ^3^ Graduate School of Advanced Science and Engineering Waseda University 3‐4‐1 Okubo Shinjuku‐ku Tokyo 169‐8555 Japan

**Keywords:** cellular stiffness, cytoskeletal crosstalk, ECM chemical cues, microtubules

## Abstract

The extracellular matrix determines cell morphology and stiffness by manipulating the cytoskeleton. The impacts of extracellular matrix cues, including the mechanical and topographical cues on microtubules and their role in biological behaviors, are previously studied. However, there is a lack of understanding about how microtubules (MTs) are affected by environmental chemical cues, such as extracellular matrix density. Specifically, it is crucial to understand the connection between cellular morphology and mechanics induced by chemical cues and the role of microtubules in these cellular responses. To address this, surfaces with high and low cRGD (cyclic Arginine‐Glycine‐Aspartic acid) peptide ligand densities are used. The cRGD is diluted with a bioinert ligand to prevent surface native cellular remodeling. The cellular morphology, actin, and microtubules differ on these surfaces. Confocal fluorescence microscopes and atomic force microscopy (AFM) are used to determine the structural and mechanical cellular responses with and without microtubules. Microtubules are vital as an intracellular scaffold in elongated morphology correlated with low cRGD compared to rounded morphology in high cRGD substrates. The contributions of MTs to nucleus morphology and cellular mechanics are based on the underlying cRGD densities. Finally, this study reveals a significant correlation between MTs, actin networks, and vimentin in response to the underlying densities of cRGD.

## Graphical abstract

Here, bio‐inert surfaces are used as a platform to study the relationship between cellular morphology and mechanics, which are influenced by chemical cues and microtubules. These surfaces will bear different densities of high and low ECM‐derived peptides (cRGD) as a chemical cue to investigate how microtubule disruption alters these cellular responses.

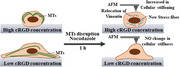

## 1 Introduction

The cellular cytoskeleton comprises three systems: actin, microtubules (MTs), and intermediate filaments. These systems significantly contribute to cellular functions, including cell adhesion, migration, and mechanical properties. MTs, in particular, are a vital part of the cytoskeleton as they are involved in essential biological processes such as cargo transport,^[^ ^1^ ^]^ cell division,^[^ ^2^ ^]^ cell movement,^[^ ^3^ ^]^ axonemal beatings,^[^ ^4^, ^5^ ^]^ and cellular mechanics.^[^ ^6^ ^]^ Despite being stiff polymeric tubes, the MT's contributions to cellular mechanics are still a matter of debate. As there are certain conditions where MTs have been observed to directly contribute to cellular stiffness; in other situations, they do not seem to have any impact. For instance, when osteoblast‐like cells are cultured in fibronectin, disruption MTs don't significantly affect cellular stiffness instead, actin plays a major role in determining stiffness.^[^ ^7^ ^]^ Upon the disruption of MTs, there is no alteration in the cellular stiffness that could be observed for porcine coronary arteries.^[^ ^8^ ^]^ MTs do not contribute to the stiffness of normal cells while their contribution is observed for cancer cells.^[^ ^9^ ^]^ Other work supported the notion that MTs alter the cells' mechanical stiffness due to their alteration of the cellular actomyosin contractility.^[^ ^10^, ^11^ ^]^ When MTs are disrupted, the deeper parts of cells become softer, while the stiffness of the peripheral cellular regions remains unchanged.^[^ ^12^ ^]^ These contradictory results could be attributed to the use of different cell types.^[^ ^9^ ^]^ However, MTs are dynamic structures and can readily change depending on the cellular requirements that vary in different situations, as well as among different cell types. The needs of a cell can vary based on its surroundings, such as the extracellular matrix (ECM). The ECM provides the structural, mechanical, and biochemical signals that can change how the cell functions and what it needs. Therefore, how the ECM cues alter the MTs has recently grabbed the attention despite the lack of physical association between MTs and ECM either directly or through other binding proteins. In fact, there is a gap of tens of nanometers between the MTs and assembled focal adhesions (FA) that directly connect to the ECM.^[^ ^13^ ^]^ Nevertheless, the mechanical^[^ ^14^, ^15^, ^16^, ^17^, ^18^, ^19^ ^]^ and topographical cues^[^ ^20^, ^21^ ^]^ of the extracellular matrix (ECM) play an essential role in shaping the characteristics of microtubules (MTs) and their involvement in cellular processes such as shape changes, morphogenesis, migration, and adhesion. Especially, the regulation by ECM biochemical cues has been studied from early years. For example, the increase in fibronectin density has been associated with increased polymerization of tubulin and the formation of MTs.^[^ ^14^ ^]^ This was observed for other ECM proteins, such as laminin and collagen.^[^ ^22^ ^]^ Moreover, in 3D mammary culture model the proper MT organization is necessary to apicobasal polarity and lumen formation, this MT organization is correlated to the interaction of β_1_ integrin with ECM proteins.^[^ ^23^ ^]^ These reactions were highly dependent on the type of ECM. Also, laminin aids in the polarization of neurons by regulating the directional assembly and stabilization of microtubules through the β1 integrin.^[^ ^24^ ^]^ The accumulation of RNA by MTs at invasive ends correlates with laminin, promoting 3D collective cell invasion.^[^ ^25^ ^]^

Regarding the regulation by ECM biochemical cues, some studies reported time‐dependent changes in the MT responses. For example, during the initial spreading process, the laminin caused an increase in the total mass of microtubules (MTs), followed by a subsequent decrease.^[^ ^22^ ^]^ Another study reported that laminin promoted the plus‐end microtubule assembly in neuronal cells.^[^ ^24^ ^]^ However, most of these studies relied on the physically adsorbed proteins, which are susceptible to ECM remodeling by exchange adsorption of serum proteins and crosslinking/digestion by oxidases/proteases. This can make it difficult to interpret whether the observed time‐dependent processes are intrinsic microtubular characteristics of cells or the time‐dependent changes in the ECM biochemical cues caused it. Moreover, the native ECM proteins have multiple binding motifs with the redundancy of cellular integrins; this made it complex to estimate the available ECM ligands during cell‐substrate interaction. In this regard, utilizing a bio‐inert surface bearing an ECM‐derived peptide provides a promising platform to investigate cellular responses' dependence on the ECM‐derived biochemical cues. In fact, we have earlier demonstrated that epithelial cells underwent early epithelial‐mesenchymal transition (EMT) just by reducing the surface density of biochemical cues by using a gold substrate functionalized with an ECM‐derived cyclic RGD peptide (cRGD) and bioinert hexa(ethylene glycol) (EG_6_).^[^ ^26^ ^]^ Due to the presence of bioinert EG_6_, we have successfully correlated the surface cRGD density and cellular responses, such as time‐dependent changes in epithelial and mesenchymal markers, as well as cellular morphological changes.^[^ ^26^ ^]^ Moreover, we were able to associate these cellular behaviors with the changes in the degree of clustering of integrin α_v_β_3_. By the use of a photoactivatable polyethylene glycol group, these gold substrates functionalized with various cyclic RGD densities, and EG_6_ showed an alteration in leader cell appearances and collective cell migration.^[^ ^27^ ^]^ Here, we utilized the same cRGD‐tethered surface with various ligand densities to investigate how MTs were involved in the epithelial/mesenchymal morphological response associated with the changes in the density of biochemical cues. In addition to the EMT‐like morphological transitions discussed, we focused on the nuclear morphological and cellular stiffness changes, which are essential mechanobiological responses. To clarify the involvement of MTs in the cellular, structural, and mechanical responses, we chemically disrupted MTs by using nocodazole. We investigated whether MTs are critical to determining cellular/nuclear morphologies and mechanics. Our results clearly showed a high dependence on MTs' contribution to the cellular responses on the ECM biochemical cues and close crosstalk between MTs and actin networks and vimentin.

## 2 Results and Discussion

### 2.1 cRGD Ligand Density Alters Actin and Microtubules Cytoskeletal System Spatial Arrangement

**Figure 1:**
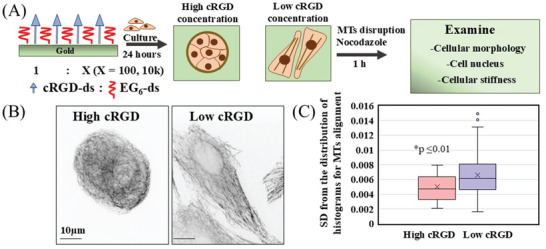
Different cRGD ligand densities are associated with alteration in cytoskeletons. A) Scheme outlines the composition of high and low cRGD‐coated surfaces, as well as the experimental flow used in the study. B) IF staining of MTs for MDCK cells cultured in these surfaces (60X magnification). C) The standard deviation between histogram distributions for MTs alignment in each cell cultured in high and low cRGD‐coated surfaces (*n* = 46 cells), Student's *t*‐test (^*^ *p* ≤0.01).

In this study, we used gold substrates functionalized with disulfide compounds bearing cell‐adhesive cRGD peptide and hexa(ethylene glycol) (EG_6_) at the end.^[^ ^26^ ^]^ By changing the mixing ratio of the two disulfides, we are able to control the surface density of the cRGD ligand (**Figure** **1**A). Specifically, we mainly used two different cRGD density surfaces, high cRGD (cRGD: EG_6_ = 1:100) and low cRGD (cRGD: EG_6_ = 1:10 000). Hereafter, we call these surfaces high cRGD and low cRGD surfaces, respectively, for simplicity. To examine how the cells feel the different cRGD, we observed the differences in focal adhesions of cells cultured on surfaces coated with high and low cRGD. We used vinculin immunofluorescence staining (as shown in Figure S1A, Supporting Information) to visualize these differences. The focal adhesion appeared as small and dispersed points with equal areas within the cells cultured on all surfaces (Figure S1B, Supporting Information). However, we noticed that the number of focal adhesions varied between the two surfaces. The cells cultured on high cRGD‐coated surfaces exhibited a significantly higher number of focal adhesions per unit area compared to those cultured on low cRGD‐coated surfaces (Figure S1C, Supporting Information). This suggests that alteration in cRGD ligand density did not affect the area of FAs but rather its numbers. As reported in our previous study, MDCK cells cultured on the high cRGD surface showed the distribution of actin in the form of peripheral actin bundles with nearly spherical epithelial morphologies (Figure S1D, Supporting Information). In contrast, the cells cultured on the low cRGD surface showed the formation of stress fibers with more spreading mesenchymal morphologies (Figure S1D, Supporting Information). In addition to these significant changes in the actin distribution, that of MTs changed drastically. Figure 1B shows the immunofluorescence images of MTs. On the high cRGD surface, MTs showed radiated organization with bending ends, while on the low cRGD surface, the microtubules distributed parallel to the long axis of the extended cells. Next, to quantitatively evaluate the change in the alignment of MTs. We analyzed the alignment of MTs in cells cultured on high and low‐cRGD‐coated surfaces. We found parallel alignment of MTs in low cRGD‐coated surfaces, while high cRGD‐coated surfaces showed net‐like structures of MTs (Figure 1C). Therefore, the spatial arrangements of MTs are highly dependent on the density of underlying cRGD ligands. We can conclude that cells integrate these different adhesive ligand densities and respond to them by alterations in cytoskeletal organization, including MT spatial distributions.

### 2.2 The Alteration in Cellular Morphology and Actin Cytoskeleton upon the Loss of MTs

**Figure 2:**
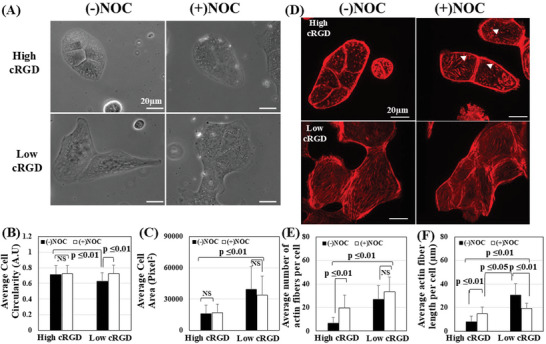
Nocodazole treatment alters the morphology and actin cytoskeleton. A) Phase contrast images of MDCK cells with and without the treatment with nocodazole for 1 h. (20X magnification). B) The calculated cellular circularity of MDCK cells with and without nocodazole cultured in high cRGD and low cRGD‐coated surfaces (*n* = 35–104 cells). C) The calculated cellular area did not change for MDCK cells with and without nocodazole cultured in high cRGD and low cRGD surfaces (*n* = 35–104 cells) (1 pixel = 0.323 μm). D) IF staining of F‐actin (Red) for MDCK cells shows the appearance of small and short stress fibers (arrowheads) for cells cultured in high cRGD after nocodazole treatment. (60X magnification). E) The average length of actin fibers within a cell cultured in low and high cRGD substrates with and without nocodazole treatment (*n* = 25–20 cells). F) The average number of actin stress fibers within a cell cultured in low and high cRGD substrates with and without nocodazole treatment (*n* = 25–20 cells). All data are presented as mean ± SD, Student's *t*‐test (*p* ≤ 0.01), (*p* ≤ 0.05).

To examine if these different MT arrangements play any role in the cellular morphology induced by the different cRGD densities, the MDCK cells cultured on the high and low cRGD‐coated surfaces were incubated with nocodazole for 60 minutes at 37 °C. Nocodazole causes the disassembly and disruption of MTs. We confirmed the complete depolymerization of MTs by staining MTs after nocodazole treatment; the disappearance of assembled MTs and uniform distribution of tubulin moieties within the cells was observed (Figure S2, Supporting Information), After that, we examined the cellular morphology, circularity, and area (**Figure** **2**A–C). The cells cultured on the high cRGD substrate maintained the rounded morphology after losing MTs (Figure 2A). On the other hand, the cells cultured on the low cRGD surface have lost their mesenchymal appearance by changing their spindle‐like elongated morphology to more cubical‐like morphology (Figure 2A). This was quantitively calculated in terms of cellular circularity that showed no significant alteration for those cells cultured on the high cRGD surface (Figure 2B). In contrast, the circularity significantly increased after treatment with nocodazole for the cells cultured on low cRGD surfaces (Figure 2B). The cellular spreading area did not alter substantially for both cells after the disruption of MTs (Figure 2C). This emphasized the role of MTs in the mesenchymal morphology correlated with the low cRGD concentrations. Previously, the contribution of MTs in cellular morphology was reported to be cell‐type dependent.^[^ ^28^ ^]^ Therefore, we conclude that the interplay and crosstalk between ECM biochemical cues and cytoskeletal systems determine cellular morphology regardless of their types. Our results indicate that MTs play a significant role in such crosstalk as an intracellular structural scaffold in the elongated morphology correlated with the low cRGD density, while the existing actin stress fibers alone are not enough to support this morphology. In cases of rounded cell shape on high cRGD surface, where there are actin bundles on the outer edges, microtubules do not play a role in shaping the cellular morphology. Next, we examine the status of actin stress fibers after the depolymerization of MTs. Figure 2D shows the F‐actin with and without the NOC treatment. The quantitative analysis revealed a notable increase in the number (Figure 2E) and length (Figure 2F) of stress fibers in cells cultured on a high cRGD‐coated surface after NOC treatment compared to the control cells. On the high cRGD‐coated surface, the actin is redistributed from the peripheral bundles to form short‐stress fibers after the nocodazole treatment. In contrast, for cells cultured on the low cRGD surface, we could not observe any significant change in the number of existing stress fibers with NOC treatment (Figure 2E). These stress fibers appeared shorter due to the loss of extended phenotype after treatment with nocodazole (Figure 2F). According to the literature, the depolymerization of microtubules (MTs) can lead to the release of signaling molecules that are responsible for forming stress fibers, such as GEF‐H1,^[^ ^29^ ^]^ this activation of GEF‐H1 can, in turn, activate RhoA which is responsible for the polymerization of actin stress fibers,^[^ ^30^, ^31^ ^]^ Despite the lack of direct experimental evidence, this could suggest the presence of a correlation between GEF‐H1 and the formation of newly created actin fibers on high cRGD‐coated surfaces. This connection should be explored further in future studies. The reason for the appearance of new stress fibers on high cRGD compared to low cRGD could be the availability of actin from different resources. On the high cRGD surfaces, cells have used actin moieties to assemble the peripheral bundles; therefore, upon MTs depolymerization, the release of signaling molecules would cause the release of actin from bundles and their consumption to form new stress fibers. In contrast, there are limited resources of actin moieties for cells cultured on low cRGD‐coated surfaces since most of them are already consumed in forming stress fibers. This can solve the debate between reported data in the literature on which disassembly of MTs is associated with an increase^[^ ^32^, ^33^ ^]^ or no change^[^ ^34^ ^]^ in stress fiber formation. Based on our observations, it appears that the status of the actin cytoskeleton prior to MT disruption is a determining factor for the appearance of new stress fibers. To sum up, the disruption of MTs with Nocodazole caused the loss of elongated morphology induced by low cRGD density without forming new stress fibers. In contrast, new stress fibers are formed for surfaces coated with high cRGD concentration without altering epithelial morphology. This suggests that the impacts of MTs depolymerization on cellular structures and actin cytoskeleton are influenced by the underlying ligand densities of cRGD‐coated substrates.

### 2.3 MTs Disruption Affects the Nuclear Morphology in Cells Cultured on High cRGD‐Coated Surfaces Compared to Low cRGD‐Coated Surfaces

**Figure 3:**
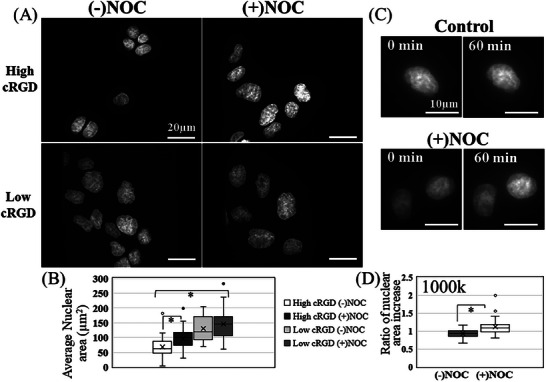
Nocodazole treatment alters the nucleus in various cRGD‐coated surfaces. A) IF staining of the nucleus for MDCK cells with and without nocodazole cultured in high cRGD and Low cRGD‐coated surfaces (40X magnification). B) The change in nuclear area for cells cultured in high cRGD after nocodazole treatment (*n* = 24–57 cells). C) IF staining of the nucleus for MDCK cells cultured in very low adhesive surface (cRGD: EG6 1:1000k) and after 60 min with and without nocodazole treatment (60X magnification). D) The average nuclear area increases after 60 minutes with nocodazole treatments, while this was not observed for control (*n* = 24–34 cells). Student's *t*‐test (*p* ≤0.01).

Here, we aimed to investigate the impact of MTs disruption on the nucleus for cells cultured on low and high cRGD‐coated surfaces. This study is particularly relevant since microtubules are a rigid framework that helps to maintain the cellular structural equilibrium and protect against nuclear deformation due to environmental and mechanical cues such as substrate stains^[^ ^35^ ^]^ and stiffness.^[^ ^36^ ^]^ Beyond this, MTs can directly affect the nucleus shape by applying force through molecular motor proteins at MTs‐minus ends.^[^ ^37^, ^38^ ^]^ For instance, the interactions between MTs and the nuclear envelope mainly cause invagination of the nucleus and altered chromatin condensation.^[^ ^39^ ^]^ These interactions contribute to the nuclear shapes of eukaryotes.^[^ ^40^ ^]^ We investigated the changes in the shape of the nucleus for cells cultured in various cRGD densities by comparing the nuclear area with and without the use of Nocodazole. **Figure** **3**A shows the Hoechst‐stained nucleus for cells cultured on high and low cRGD‐coated surfaces. For the high cRGD coated‐substrate, cellular nuclei appeared rounded and smaller in size (Figure 3B). This contrasts the nucleus of cells cultured on the low cRGD surface, where the nuclei have larger areas (Figure 3B). Therefore, we observed different nuclear shapes for cells cultured on these various cRGD‐coated surfaces. Since actomyosin structures play a significant role in modulating nuclear shape,^[^ ^41^, ^42^ ^]^ The appearance of parallel actin stress fibers during cell spreading and elongation on low cRGD coated surfaces generates the tensile force required for nuclear compression toward the substrate, so larger nuclear areas would be observed. In the case of the isotropic contractile peripheral actomyosin bundles observed for cells cultured on high cRGD‐coated surfaces, they will not induce such an effect, so the formation of a smaller area and rounded nucleus is observed. Upon the MTs disruption, we could observe an increase of nuclear area for cells cultured on high cRGD‐coated surfaces despite the maintenance of the same cellular area, while this was not observed for low cRGD surfaces (Figure 3A,B). This marked increase in the nuclear area after the vanishing of MTs could be due to the appearance of new stress fibers, which increase the tensile stress, which causes the pulling of the nucleus to the substrates. The apical stress fibers pushing the nucleus down may not influence the observed change in the nucleus area since the newly appeared stress fibers are not sufficiently long to pass over the nucleus and connect at their two ends to substrates (Figure S3, Supporting Information). There is another possibility that microtubules (MTs) shape the nucleus on high cRGD‐coated surfaces differently. It has been proved that MTs can influence the geometry of the nucleus^[^ ^38^, ^39^ ^]^ by causing its invaginations at the site where the MTs are polymerizing. This process depends on the MTs and nuclear lamin A.^[^ ^39^ ^]^ In the case of MDCK, MT polymerization occurs in various positions around the nucleus rather than from a single spot, like the pair of centrioles.^[^ ^43^ ^]^ Therefore, the symmetric distribution of forces is expected to result from the interactions between the MTs' multiple polymerization positions and the nucleus in MDCK cells. To test a similar cellular state, we utilized a very low adhesive surface where cRGD: EG_6_ concentration was 1:1 000 000 (1000K). On this surface, the cells maintained a circular morphology, did not spread at all, and did not form any stress fibers (Figure S4A,B, Supporting Information). Upon treatment of cells with Nocodazole, we could not observe any formation of new actin stress fibers either (Figure S4B, Supporting Information). At the same time, when we examined the nuclear size change, at 0 min and 60 min after nocodazole treatment, we could observe an increase in the nuclear area after the disruption of MTs compared to control cells (Figure 3C,D). When we checked the side view of the nucleus, we observed an increase in both the vertical and horizontal directions, rather than just one direction, as in the case of nuclear compression toward the surface (Figure S4C, Supporting Information). This increase in the nuclear area could suggest that MTs contribute to maintaining a smaller nucleus area in rounded cells where stress fibers do not determine the nuclear shape. MTs are dynamic structures that undergo continuous assembly and disassembly in plus ends near the membrane; these forces can pull and push other organelles.^[^ ^44^ ^]^ These forces could originate an opposing force near the nucleus to maintain rounded and small morphology. Thereby, we expect that in highly cRGD‐coated surfaces where actin existed in peripheral bundles, MTs could serve a dual purpose. First, a biochemical role in which MTs scavenge the signaling molecules to restrain the formation of actin stress fibers and maintain the peripheral actin bundles. In addition, there is a mechanical role involved in maintaining nuclear structures. As a result of MT disruptions, neither the smaller nuclear area nor the peripheral bundles can be preserved, resulting in the nucleus losing its smaller area and becoming larger. Meanwhile, for 10k surfaces where the actin stress fibers exist, the MTs do not contribute to forming or maintaining a larger nuclear area. Therefore, we conclude the contribution of MTs in maintaining the nuclear shape is correlated with their underlying cRGD ligand densities.

### 2.4 The Alteration in Cellular Mechanical Properties upon the Loss of MTs Depends on Underlying Substrate cRGD Density

**Figure 4:**
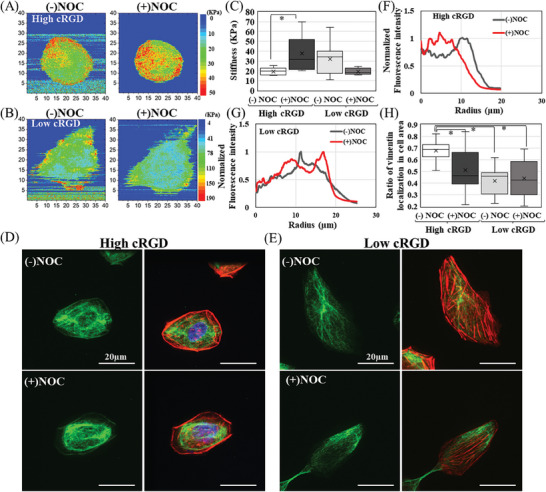
Nocodazole treatment alters the cytoskeletal stiffness on various cRGD‐coated surfaces. Elastic modulus map for the same cell cultured in A) high cRGD and B) low cRGD‐coated surfaces before and after the treatment with nocodazole. C) The average elastic modulus obtained from line scan for cells cultured in high cRGD and Low cRGD‐coated surfaces with and without nocodazole treatment (*n* = 5–10 cells). IF staining of Vimentin (green) alone and in merged photos that shows actin (red) nucleus (blue) for single cells cultured in D) high cRGD and E) low cRGD‐coated surfaces after the nocodazole treatment (60X magnification). Radial distribution profiles were calculated from cells cultured in F) high cRGD and G) low cRGD‐coated surfaces with and without nocodazole treatment. H) The ratio of vimentin localization relative to total cellular area (*n* = 8–12 cells). Student's *t*‐test (*p* ≤ 0.05) and (*p* ≤ 0.01).

Here, we have used AFM to determine the alteration of the cellular elastic modules for cells cultured on low and high cRGD‐coated surfaces, with and without Noc treatment. AFM can measure cellular mechanics with nanometric resolution in culture mediums, providing morphological and topographical measurements. We used AFM indentation on cells cultured on our surfaces. Tip radius, R, was expressed as a linear combination of a constant term r, two topographical gradient‐dependent terms of a*δz/δx and b*δz/δy, and a depth‐dependent term of c*d, where a, b, c are to‐be‐determined coefficients. A spectrum image, a 2D matrix of 256 × 256 f‐d curves, was acquired on living MDCK cells. **Figure** **4**A,B show modulus maps for a cell cultured on high and low RGD‐coated surfaces before and after nocodazole treatment. The cellular elastic modules for these cells cultured on low adhesive surfaces have higher values than those cultured on high cRGD‐coated surfaces (Figure 4A,B). It was previously reported that cortical stiffness increases as cell spreading areas increase.^[^ ^45^, ^46^ ^]^ To confirm that the observed difference is attributable to changes in cell spreading areas, we patterned single cells in a circular shape using photoactivatable PEG‐coated surfaces (PCP). We cultured MDCK cells on low and high cRGD‐coated surfaces after irradiating with PCP groups, as reported previously.^[^ ^27^ ^]^ Based on the calculated modulus, the variation in cRGD concentrations did not significantly impact cortical elasticity, given that cellular areas were fixed (Figure S5A,B, Supporting Information). Based on the findings, it can be inferred that using low cRGD substrates for the cultured cells resulted in changes in cell‐substrate adhesion, increasing the cell‐spreading area and vice versa in the case of high cRGD surfaces. This alteration in cell spreading is critical in determining the modulus for the free state.

Next, we investigated how MTs disruption affects the cellular mechanical properties in relation to the underlying substrates. The measurement started after 30–60 minutes of Noc treatment, so the transient effect of MTs disruption (5 min) was not observed.^[^ ^11^ ^]^ Intriguingly, high cRGD‐coated surfaces exhibited an increase in cellular elastic modules after MT disruption (Figure 4A), while low cRGD‐coated substrates showed a decrease (Figure 4B). Typical force curves for the cells cultured in high and low cRGD‐coated surfaces before and after the nocodazole treatment are shown (Figure S6, Supporting Information). To quantify these changes, we utilized AFM using line scanning to generate force‐distance curves and detect alterations in cellular elastic modules for several cells with and without NOC treatment (Figure 4C). We selected cells with varying degrees of cellular spreading on 10k surfaces to minimize the impact of spreading when comparing before and after NOC treatment. Notably, the increase in cellular stiffness on high cRGD surfaces after MT disruption was significant, whereas it was not statistically significant for 10k‐cultured cells when considering only cellular elastic module values. We can state that MTs for the cells cultured on low cRGD did not play any significant role in the cell stiffness. For MDCK cells on high cRGD surfaces, MTs are necessary for maintaining cellular stiffness since the interruption of MTs causes elasticity to increase. This alteration in cellular elasticity was not correlated to any change in cellular spreading areas or circularity (Figure 2B,C). The contribution of MTs in cellular mechanics is still a perplexing question since MTs have been observed to contribute inconsistently to cellular stiffness in multiple reports. For instance, upon the disruption of MTs, there is no alteration in the cellular stiffness that could be observed for porcine coronary arteries^[^ ^8^ ^]^ or fibroblasts.^[^ ^47^ ^]^ MTs do not contribute to the stiffness of normal cells, while their contribution is observed for cancer cells.^[^ ^9^ ^]^ When osteoblast‐like cells are cultured in fibronectin, disruption MTs do not significantly affect cellular stiffness.^[^ ^7^ ^]^ Other work supported the notion that MTs alter the cells' mechanical stiffness due to their alteration of the cellular actomyosin contractility.^[^ ^10^, ^11^ ^]^ These contradictory results within these studies could be attributed to cell‐specific characteristics, as Gardy and his colleagues suggested. In our case, we observed these varying results for the same cells cultured in different cRGD ligand densities, suggesting the alteration in the cell‐substrate adhesion alters the cytoskeletal systems interactions and the MTs' contribution to cellular stiffness.

This increase in cellular stiffness for high cRGD surfaces likely resulted from the newly formed short‐stress fibers. The role of actin in determining cellular stiffness is widely acknowledged^[^ ^47^ ^]^ since it is known that the organization,^[^ ^48^, ^49^ ^]^ accumulation,^[^ ^50^ ^]^ crosslinking,^[^ ^51^ ^]^ and contractile activation^[^ ^11^ ^]^ of stress fibers contribute to cellular stiffness. Although the modulus values of cells cultured in high cRGD (with NOC) are nearly equivalent to those of control cells cultured in low cRGD (Figure 4C), there are differences in the quantity, distribution, and localization of stress fibers between cells cultured on high cRGD surfaces after NOC treatment and control low cRGD surfaces (Figure 2D). This suggests that another cytoskeletal system could collaborate with actin to enhance cellular stiffness in the absence of MTs. To explore this hypothesis, we stained vimentin with and without NOC treatment. We noticed a variation in vimentin localization for high cRGD‐coated surfaces for single cells (Figure 4D–H) and cells in clusters (Figure S6A,B, Supporting Information). However, the vimentin expression levels remained unchanged (Figure S7C, Supporting Information). For cells cultured on low cRGD‐coated surfaces, we could not observe such a change in the localization after NOC treatment. The redistribution of vimentin occurs, along with newly formed actin filaments for cells cultured in the high cRGD. This could compensate for the absence of MTs on these surfaces. Vimentin plays a significant role in determining cellular mechanical properties.^[^ ^52^, ^53^ ^]^ Protecting against compressive stress, but this protection correlates with substrate stiffness.^[^ ^54^ ^]^

In this study, the cellular morphology and spreading responses to the variation in cRGD concentrations were intriguing. When cells are exposed to high cRGD‐coated surfaces, there is a tremendous increase in the number of focal adhesions per unit area, which impedes their spreading due to more significant available ligands of cRGD. On the other hand, low cRGD concentrations allow cells to spread into a mesenchymal‐like morphology. In contrast, deficient cRGD concentrations lead to cell rounding again due to the lack of adhesion spots. Understanding the biphasic relationship between cRGD concentrations and cellular behavior is essential. The soft matter models^[^ ^55^ ^]^ provide a plausible explanation for the second part of this relationship. However, it's important to note that the theory cannot be applied to the first part since our surfaces have displayed higher values of available cRGD ligands than the range observed upon coating surfaces by ECM proteins.^[^ ^56^ ^]^ The role of microtubules in spreading is minimal on the sides of the biphasic curve because there was no change in spreading when the microtubules were disrupted. In contrast, for low cRGD, MTs are the primary determinant of cellular spreading and mesenchymal morphology.

**Figure 5:**
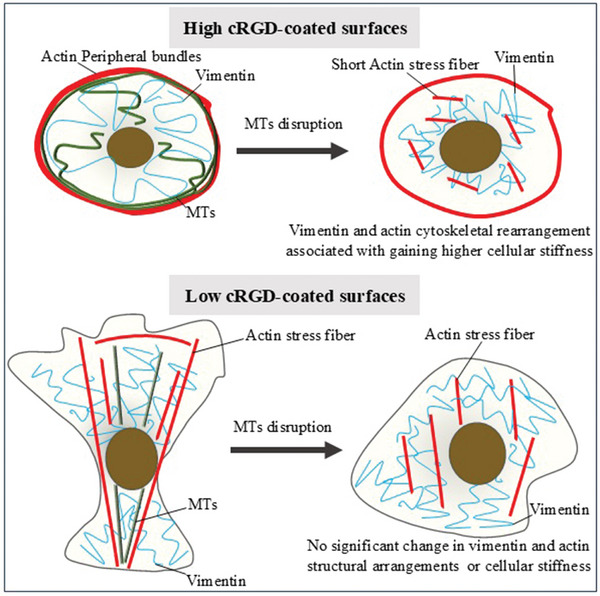
Cartoon illustrating the cellular structures with various cytoskeletal systems (MTs, actin, vimentin) in relation to cellular stiffness for cells cultured in high and low cRGD‐coated surfaces.

Regarding cellular mechanical properties, MTs provide the framework for maintaining cellular stiffness and mechanical stability on high cRGD‐coated surfaces with peripheral actin bundles, with vimentin's contribution being minimal. In the absence of MTs, vimentin redistribution and small actin stress fibers’ formation would maintain epithelial morphology, increasing cellular stiffness (**Figure** **5**). This implies that cooperation between cytoskeletal systems is necessary. Conversely, for low cRGD‐coated surfaces, the absence of MTs does not significantly alter actin or vimentin structural arrangement or cellular stiffness, indicating that MTs have a less dominant role in this context. To illustrate our results, Figure 5 shows the cytoskeletal arrangements induced by substrates with various cRGD densities in the presence or absence of MTs. Previous studies have reported varying effects of MT disruption on cellular stiffness depending on environmental cues such as serum presence,^[^ ^57^ ^]^ cell type,^[^ ^9^ ^]^ and substrate stiffness.^[^ ^58^ ^]^ Our study introduces a new environmental cue: the underlying cRGD density, which is pivotal in determining the involvement of MTs in cellular mechanical properties. Based on our findings, cellular stiffness results from the interplay between different cytoskeletal systems, and we have identified a noteworthy correlation between MTs, actin, and vimentin in determining cellular elasticity concerning the biochemical properties of the underlying substrate. Finally, by studying the interactions between various cytoskeletal systems in general and MTs in particular in relation to environmental stimuli, we can identify novel therapeutic approaches that aim to restore the proper functioning of microtubules (MTs). This would be particularly beneficial for treating diseases that involve both MT dysfunction and altered extracellular matrix (ECM) characteristics, such as cardiovascular^[^ ^59^, ^60^ ^]^ and neurovegetative diseases.^[^ ^61^, ^62^ ^]^

## 3 Conclusion

Here, we have used cRGD‐tethered surfaces with varying ligand densities to investigate the role of microtubules in cellular and nuclear morphological responses associated with changes in biochemical cue density. MTs did not significantly contribute to cellular morphology on high cRGD‐coated surfaces, unlike the nucleus morphology. Conversely, on low cRGD‐coated surfaces, MTs significantly contributed to maintaining the cellular morphology, unlike the nucleus morphology. The observed alteration in the cellular and nuclear morphology was directly associated with the emergence of new stress fibers from peripheral actin bundles in cells cultured on high cRGD‐coated surfaces. Meanwhile, for cells cultured in the low cRGD‐coated surface, actin stress fibers did not significantly change. Cellular elastic modules for cells cultured on high and low cRGD‐coated surfaces are dependent on the cellular spreading and associated cytoskeletal arrangements rather than solely the underlying cRGD densities. Upon disruption of microtubules, the elastic properties of cells increase when cultured on surfaces with high cRGD coating. This increase is associated with the rearrangement of vimentin and the appearance of new stress fibers. However, for cells cultured on surfaces with low cRGD coating, the elastic properties of cells remain the same, and both vimentin and actin stress fibers remain unchanged. This highlights the complex interplay between these cytoskeletal components and their role in various biological processes.

## 4 Experimental Section

### Preparation of Different Concentrations of cRGD‐Coated Gold Surfaces

Gold substrates were prepared as previously reported.^[^ ^26^, ^63^ ^]^ A thin layer of 5‐nm titanium layer followed by a 20‐nm gold layer was consecutively deposited on the glass surface (0.25 mm thick, Matsunami, Osaka, Japan) under vacuum using an E‐beam evaporator. A UV‐ozone cleaner (UV253; Filgen, Nagoya, Japan) was used to clean the gold substrates. The cleaned substrates were then incubated for two h at 25 °C with a mixed solution of disulfides in a specified ratio for 100 = (9.99:0.01) and 10k = (9.9999:0.0001), 1000k = (9.999999:0.000001), 1kk = (9.9999999:0.0000001)of EG_6_‐ds, and cRGD‐ds respectively. The starting concentration of the ligands was 50 µm. The gold substrates were washed 2–3 times with methanol and sterilized by incubation with 70% alcohol for 5 min. Then, it would be used directly as a substrate for cell culture. These concentrations were selected based on the previous comprehensive study that showed the impact of varying concentrations of cRGD diluted with EG_6_ on the behavior and fate of cells.^[^ ^26^ ^]^

### Cell Culture and Immunostaining

MDCK cells (RCB0995, RIKEN cell bank) and MDCK cells stably expressing lifeact‐green fluorescent protein (GFP)^[^ ^64^ ^]^ were cultured in MEM (Sigma, St. Louis, MO, USA) containing 10% FBS (heat‐inactivated FBS; BioWest, EU origin), 100 units/mL penicillin and 100 mg mL^−1^ streptomycin (Nacalai, Japan), 1% MEM‐nonessential amino acids (Nacalai, Japan), 1% sodium pyruvate (Nacalai, Japan), and 1% L‐glutamine (Nacalai, Japan) at 37 °C in a humidified atmosphere containing 5% CO_2_ at 75% confluency of cell subculture. Cells were collected by trypsin/EDTA (Wako, Japan) to be seeded on gold surfaces. The cell passages were used between 6 and 19 during the course of the experiments. Cell seeding occurred first in (‐) FBS medium for 1–2 h, and then the medium was changed into a complete medium for the rest of the experiments. After 18 h. of culture, Nocodazole (Abcam, AB120630) was dissolved in DMSO and added at concentration 50 µm for one h, DMSO final concentration in experiment ≤0.1%. Phase contrast images were captured by an Olympus microscope (IF81‐PAFM, Olympus, Tokyo, Japan), and a cooled CCD camera, Retiga EXi (QImaging), was used for image capturing. All systems were controlled using Metamorph software (Molecular Devices, Sunnyvale, CA, USA); captured images were processed using Fiji (Image J, USA). The circularity and area of cells cultured on a variety of cRGD‐coated surfaces were analyzed using the image processing software Fiji. The cells were manually outlined, and the circularity and area of cells were computed automatically using the area and shape descriptor under the measure function in Fiji. Confocal images were obtained under the same Olympus microscope by using a disk‐scan unit (CSU‐10, Yokogawa, Tokyo, Japan) and Andro CCD camera (SONA 4BV6U, UK), captured images were processed using Fiji (Image J, USA). For immunofluorescence staining, cells were fixed with 4% paraformaldehyde (Nacalai, Japan) for 15 min, quenched with 5% glycine (Wako, Japan) in PBS for 5 min, permeabilized with 0.5% Triton X‐100 for 5 min, and blocked with bovine serum albumin (BSA, Wako, Japan) for 30 min; cells were then incubated with Alexa Fluor 555 Phalloidin (ThermoFisher Scientific, USA) for one h. Rat monoclonal tubulin antibody (clone YL1/2, Abcam, Ab6160, 1:1000 dilution), Mouse monoclonal vimentin antibody (clone VIM 3B4, Sigma‐Aldrich; 1:150 dilution) and anti‐mouse IgG Alexa Fluor 488 (1:1000) (Life Technologies, Eugene, OR, USA), anti‐rat IgG Alexa Fluor 488 (1:1000) (Life Technologies, Eugene, OR, USA) were used. Mouse anti‐vinculin (1:400, Sigma), anti‐mouse IgG Alexa Fluor 488 (1:1000, ThermoFisher Scientific, USA), and Hoechst 33342 (1:1000, Life Technologies, Eugene, OR, USA). Fiji's Analyze particle function was used to calculate the size and number of focal adhesions formed automatically. In detail, the first step to reduce noise and enhance the photo was done by applying a convolve filter to the original photos using the process function Filters – Convolve filters. Then, areas and numbers of FAs were automatically computed by setting the measurements to area and shape descriptor functions under the measure function chosen in Fiji. Finally, Analyze particle was chosen. Areas that were <0.1 µm were excluded. To calculate the fluorescence intensities for MTs and vimentin, each cell was manually outlined, and then the integrated density was calculated by selecting the integrated density option under the measurement function in Fiji. After that, the background was subtracted, where the background represents the cell area multiplied by the mean grey value of the non‐fluorescent background.^[^ ^26^ ^]^ To calculate the change in the vimentin localization, the vimentin‐positive region was manually selected using Fiji; these areas were divided by the whole cellular areas. The distribution of vimentin within cells was analyzed by calculating its radial distribution. To do this, the radial profile plugin was utilized, as previously described.^[^ ^65^ ^]^ This plugin generated a profile plot of normalized integrated intensities around concentric circles, which showed the relationship between the distance from a point in the image and the intensity of vimentin. To calculate the alignment of MTs within each cell, the Fiji‐directionality plugin was used, which uses the local gradients orientation method.^[^ ^19^, ^66^ ^]^ This method generated a histogram of directionality for all microtubules (MTs) within each cell. The SD between the columns of histograms was calculated; a completely flat histogram represents an isotropic orientation of microtubules. A smaller standard deviation represents a flatter histogram (Figure S8, Supporting Information). For Live staining of the nucleus, Hoechst 33342 (Invitrogen, Eugene, OR, USA) was incubated with cells cultured on very low cRGD‐coated surfaces (1000k) for 7 minutes, then washing steps occurred by replacing half of the medium with a new medium, this process was repeated 3–4 times. The confocal microscope was used to observe the nucleus immediately after it was stained and washed. Afterward, NOC was added with a concentration of 50  µm. After 60–90 minutes, the imaging of the nucleus was repeated. To calculate the nucleus's area, Fiji was used to threshold the images. The area was then calculated using the “measure particle” function. For the nuclear area of 1000k surfaces, the nucleus was manually outlined before and after the NOC treatment. The ratio of area increase was then calculated by dividing the nuclear area after treatment by the nuclear area before treatment. The student's *t*‐test was performed to determine the statistical significance of all calculated data. The student's *t*‐test was performed by Excel to determine the statistical significance of all calculated data, significant *p* ≤0.05%.

### AFM Indentation

Cell cultures on gold substrates immersed in full medium were directly characterized by AFM (Park Systems, NX10) using gold‐coated cantilevers with pyramidal tips (PPP‐CONTSCAuD, NANOSENSORS) with spring constant in the 0. 1‐0.4 N m^−1^ range. All measurements were done at room temperature. The spring constant was calibrated using the thermal noise method before every experiment. At each indentation location, the tip pressed into the cell until a set force threshold value was reached. The threshold for mapping was set at 1.5 nN. Calculation of modulus was performed as previously reported.^[^ ^65^ ^]^ The study considered the actual tip shape's deviation from an ideal hemisphere by treating tip radius R as a linear combination of a constant term r and specimen‐dependent terms, which are topographic gradient in x direction, δz/δx; gradient in y direction, δz/δy; and deformation, d. The format of Tip shape expression $R\;=\;r+a\frac{\partial Z}{\partial x}+b\frac{\partial Z}{\partial y}+cd$ is was adopted. The coefficients a, b, c, and d were then generated by the Markov‐chain Monte Carlo method to provide a trial R‐value. Modulus E was best fitted using equation: force expression $F\;=\;4\pi \mathrm{Rd}\sigma \cos\;\left[tan^{-1}\left(2\;\sqrt[{}]{\frac{R}{d}}\right)\right]+\frac{4}{3(1-\gamma^{2})}ER^{0.5}d^{1.5}$ at each indentation location during one map scan. Fitting errors at each indentation were also calculated, and their variance from the entire map could be obtained. The variance was then used as feedback to the Monte Carlo algorithm for generating the following combination of a, b, c, and d for 1000 loops till minimization of variance was achieved. The resulting R is considered to be the actual radius of the tip during the map acquisition because the location dependency of fitting error should be minimal. The σ (stress) and E (modulus) obtained with this R‐value were used to produce the σ and E maps used for analysis. For average modulus calculations, AFM line scanning was performed along the centerline of the cell, as shown in the height profile (Figure S9, Supporting Information). The lowest location in the height profile is the substrate, while the tallest location is the nuclei. To eliminate the influence from the substrate, the average modulus was calculated only from the range of nucleus and cytoplasm (in ≈21 µm). The average modulus was taken from all points of the line scan; outliers were removed using Inter Quartile range calculations. The student's *t*‐test was performed to determine the statistical significance of all calculated data.

## Conflict of Interest

The authors declare no conflict of interest.

## Supporting information



Supporting Information

## Data Availability

The data that support the findings of this study are available from the corresponding authors upon reasonable request.
