# Supplementary material for: Microtubules Disruption Alters the Cellular Structures and Mechanics Depending on Underlying Chemical Cues
Source: Small. 2024 Sep 29;21(13):2312282. doi: 10.1002/smll.202312282 (PMC11962689; doi:10.1002/smll.202312282)
Supplement: Supplementary file 1 — Supporting Information [file SMLL-21-2312282-s001.pdf]

NANO · MICRO
small

Supporting Information

for *Small*, DOI 10.1002/smll.202312282

Microtubules Disruption Alters the Cellular Structures and Mechanics Depending on Underlying Chemical Cues

Shimaa A. Abdellatef, Hongxin Wang and Jun Nakanishi**

Microtubules Disruption Alters the Cellular Structures and Mechanics Depending on Underlying Chemical Cues.

Shimaa A. Abdellatef [1],Hongxin Wang[1] , Jun Nakanishi[1, 2, 3]**

[1] Research Center for Macromolecules and Biomaterials, National Institute for Materials Science (NIMS), 1-1 Namiki, Tsukuba 305-0044, Japan.

[2] Graduate School of Advanced Engineering, Tokyo University of Science, 6-3-1

[3] Graduate School of Advanced Science and Engineering, Waseda University, 3-4-1 Okubo, Shinjuku-ku, Tokyo169-8555, Japan.

*Corresponding Authors: nims.email.shimaa@gmail.com ;NAKANISHI.Jun@nims.go.jp

Keywords: ((microtubules, cellular stiffness, ECM chemical cues, cytoskeletal crosstalk))

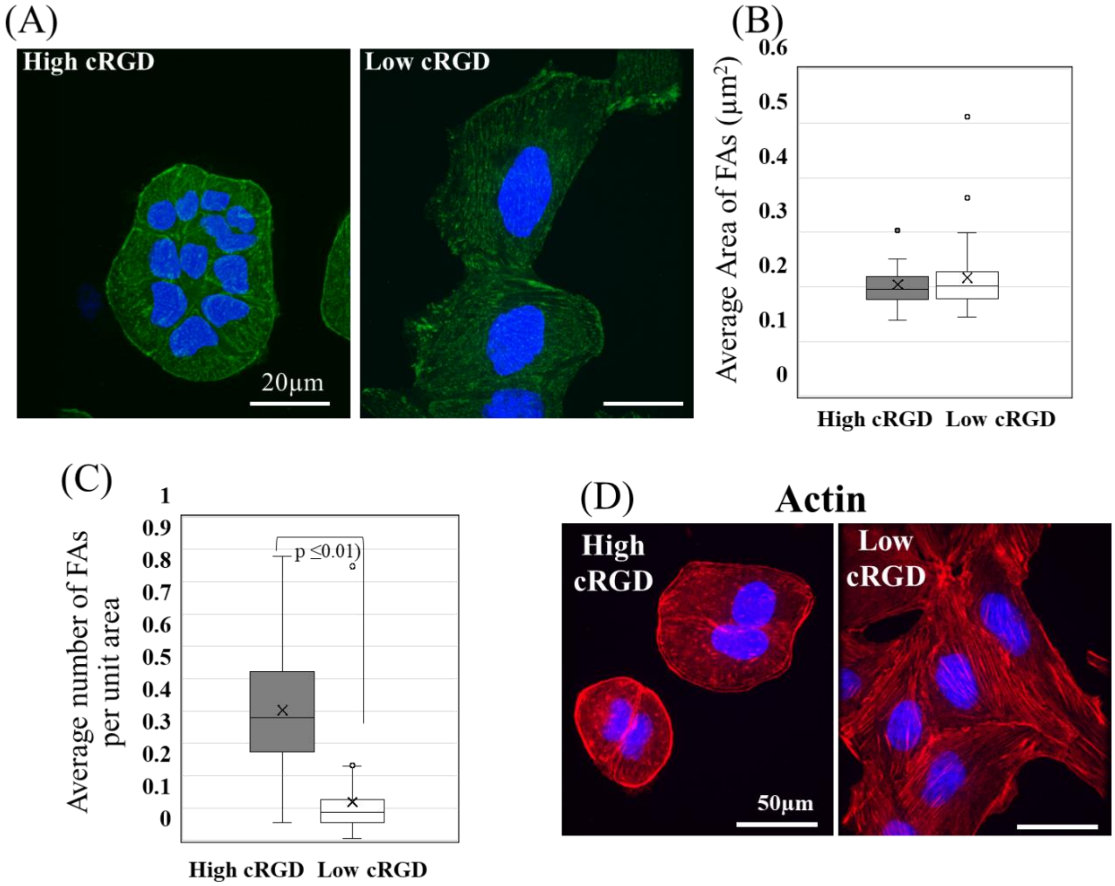

Figure S1: (A) IF images for vinculin staining (green) and nucleus (blue) to show the difference in formed focal adhesions for cells cultured in high and low cRGD coated surfaces (B) Average area of focal adhesion formed (n= 34～35 cells). (C) Average number of focal adhesions formed per unit area (n= 34～35 cells). (D) Immunofluorescence (IF) staining of F-actin (Red) and nucleus (Blue) for MDCK cells cultured in high and low cRGD-coated surfaces (60X magnification).

(A)

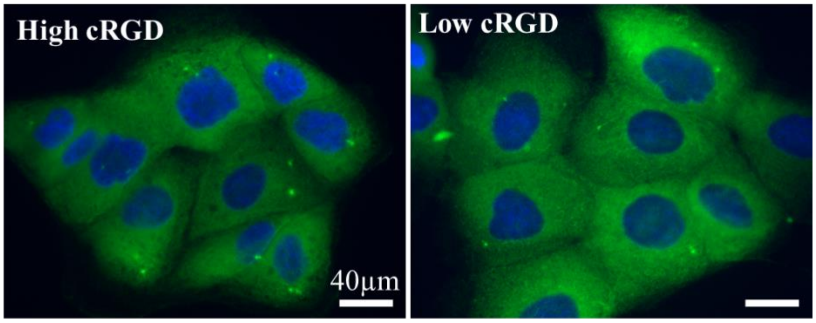

Figure S2: (A)IF staining of tubulin (green) and nucleus (blue) that shows the complete disassembly of MTs after the Nocodazole treatments.

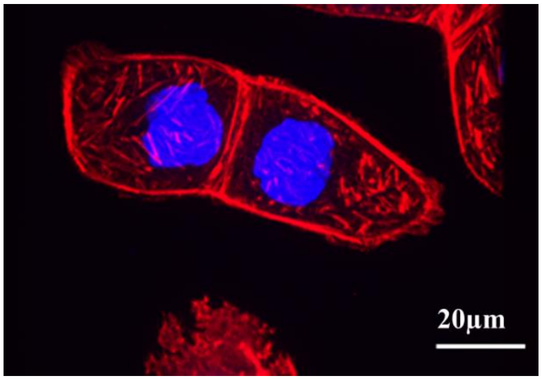

Figure S3 shows the combined IF staining of actin (Red) and nucleus (blue) for MDCK cells cultured in high cRGD coated surfaces after NOC treatment, on which short-stress fibers are observed that don't pass over the nucleus.

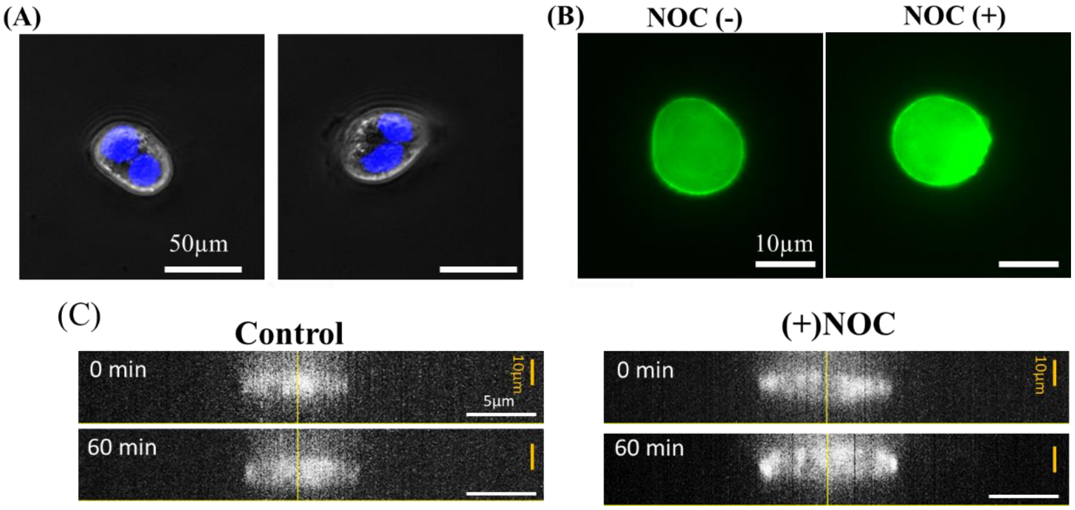

Figure S4: (A) Combined image of cells cultured (phase contrast as gray, nucleus stained with Hoechst as blue) on very weekly adhesive surfaces on which cells show rounded morphology. (B) MDCK-ACT life- cultured on very weekly adhesive surfaces for 18 hours, then treated with NOC for 1 h. (C) Side view of the cellular nucleus (stained with Hoechst) after 60 min of NOC treatment, which shows the increase in the nucleus volume in several directions.

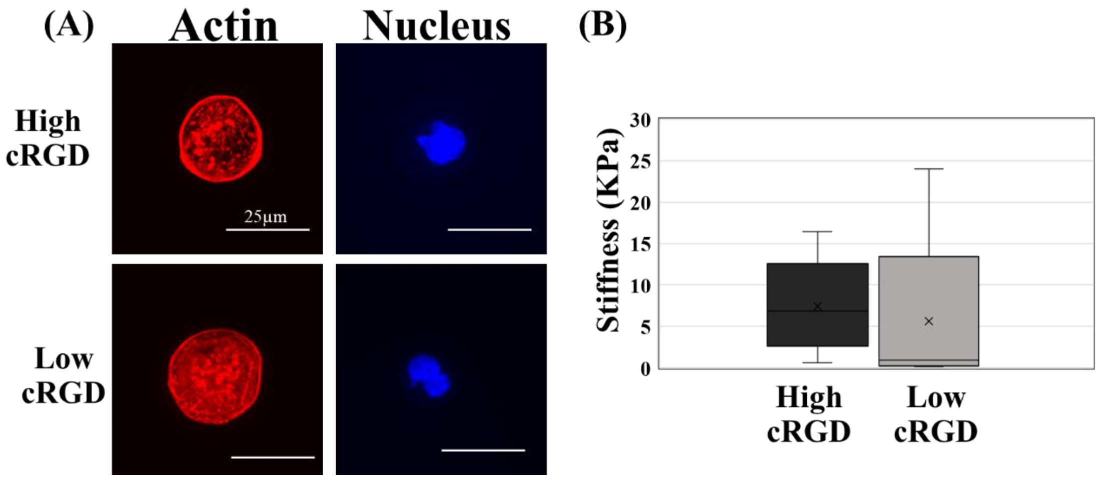

Figure S5: (A) IF staining of F-actin (Red) for MDCK cells cultured in a circular shape d=25µm using the photoactivatable PEG surfaces (PCP) with high and low cRGD concentrations. (B) The elastic modulus for cells cultured in PCP with high cRGD and Low cRGD-coated surfaces.

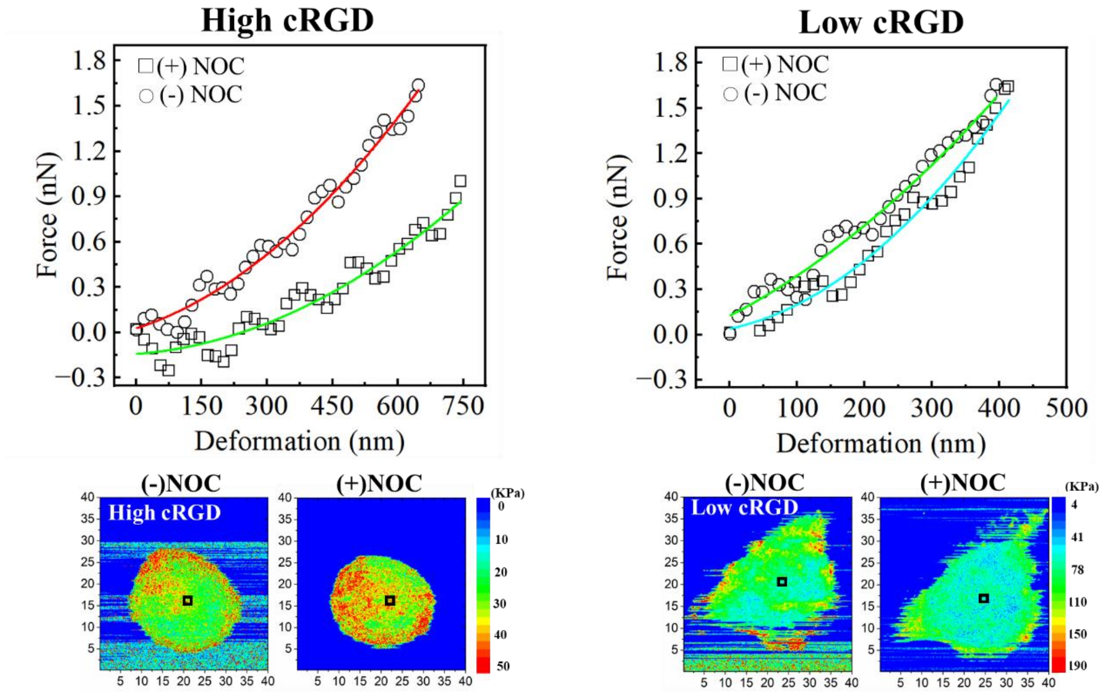

Figure S6: Force−deformation curves for cells cultured on high (A) and low (B) cRGD-coated surfaces before and after nocodazole treatment. Circular and square shapes stand for the experiment data of (+) NOC and (-) NOC respectively. On high cRGD, two fd curves were selected from the region where they were marked by black squares in the elastic modulus map. The red and green lines mean the fitting curves for (+) NOC and (-) NOC by the multivariate mode. In low cRGD-coated surfaces, two fd curves were selected from the region where they were marked by black squares in the elastic modulus map. The green and blue lines mean the fitting curves for (-) NOC and (+) NOC by the multivariate mode.

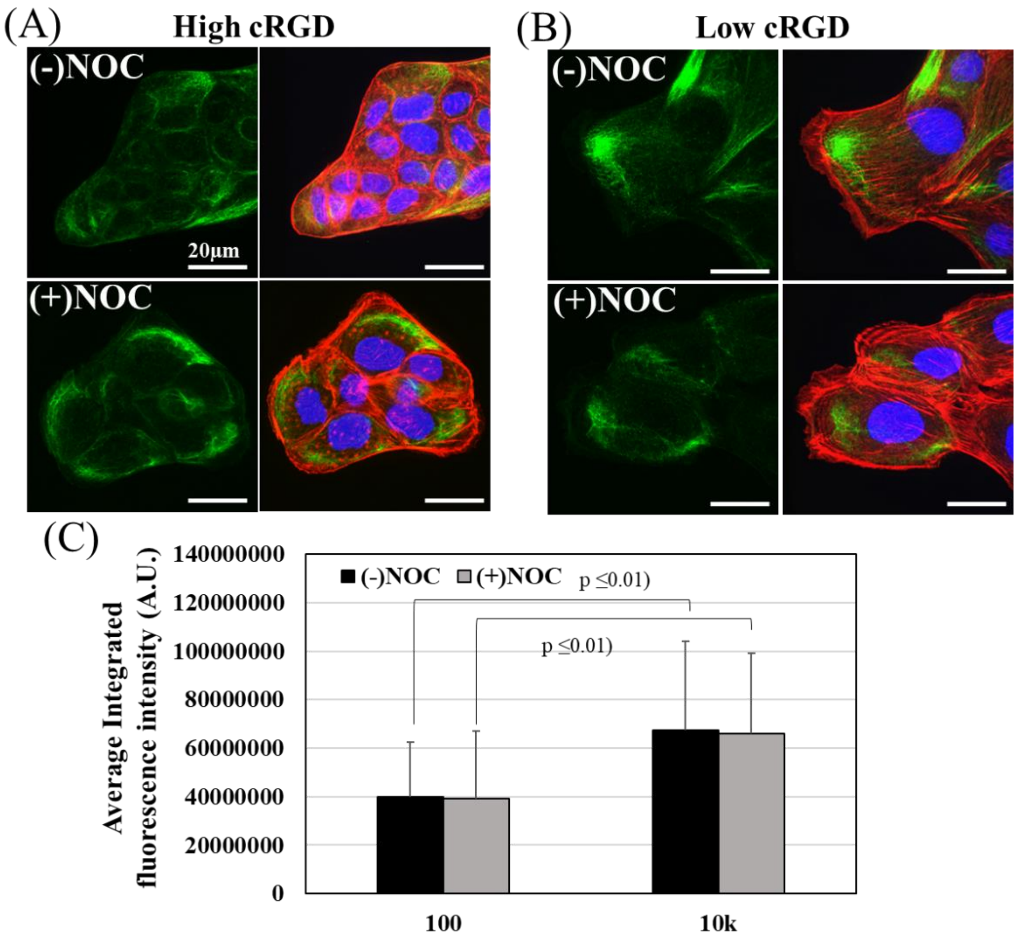

Figure S7: IF staining of Vimentin (green) alone and in merged photos that shows actin (red) nucleus (blue) for a group of cells cultured in (A) high cRGD and (B) Low cRGD-coated surfaces with and without the nocodazole treatment. (C) The calculated fluorescence intensity of vimentin localized in cells cultured in high and low cRGD substrates with and without NOC treatments (N=30 ～ 32 cells).

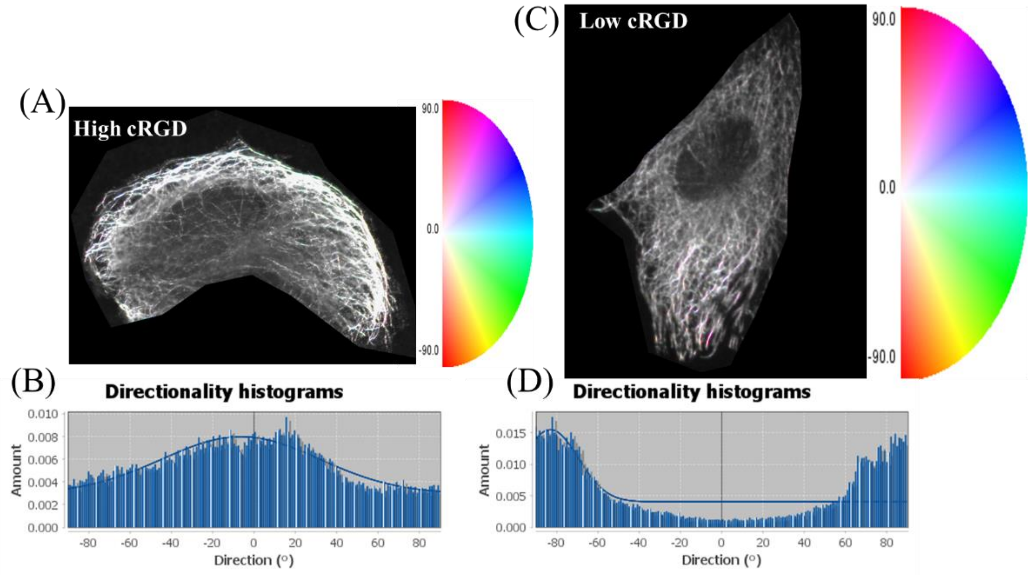

Figure S8: Shows the color maps and (A, C) directional histograms (B, D) for MTs cultured on high and low cRGD-coated surfaces, respectively, using the Fiji-directionality plugin.

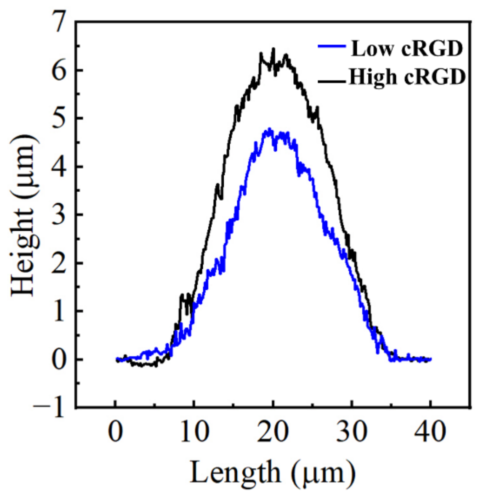

Figure S9: The height profile of a cell cultured in high and low cRGD coated surfaces obtained during AFM line scanning.
